# From Cubist Simultaneity to Quantum Complementarity

**DOI:** 10.1007/s10699-016-9494-7

**Published:** 2016-05-12

**Authors:** Christophe Schinckus

**Affiliations:** School of Management, University of Leicester, Leicester, UK

**Keywords:** Cubism, Quantum physics, Simultaneity, Bergsonian durée

## Abstract

This article offers a contribution to the history of scientific ideas by proposing an epistemological argument supporting the assumption made by Miller whereby Niels Bohr has been influenced by cubism (Jean Metzinger) when he developed his non-intuitive complementarity principle. More specifically, this essay will identify the Bergsonian durée as the conceptual bridge between Metzinger and Bohr. Beyond this conceptual link between the painter and the physicist, this paper aims to emphasize the key role played by art in the development of human knowledge.

## Introduction: Cubism and Science

Cubism is usually associated with a movement in art in which the single viewpoint perspective has been abandoned. Actually, the term “Cubism” refers to a heterogeneous movement that was composed by “several Cubisms”: the Gris’s Cubism was different from the works of Villion or Leger whereas the Metzinger conception of Cubism was far from the way of painting of the two masters Picasso and Braque (who worked together during several years). Despite existing dissimilarities between these works, they usually appear under the same umbrella of Cubism because they all proposed an innovative way of painting the world by representing, at the same time, several dimensions of a reality/object (Fauchereau 1982). Precisely, “a cubist painting requires us to recognize that our confusion about what is implied in this new method of representation is the result of misplaced expectations […] about our own ability to identify and organize our word” (Vargish and Mook 1999 p. 34). In other words, although “Cubisms” differ in terms of colour themes, level of abstraction and materials used, they all suggest that *there is no there because it is all here* in a view where “all the points in space along the path of observation occupy the same location simultaneously” (Shlain 2007, p. 188). Such an intellectual evolution in the way of thinking the notion of representation echoed scientific debates that emerged in the early twentieth Century in physics. There exists a specific literature showing that Cubism serves very well to illustrate the conceptual shift generated by the scientific environment at that time. Among other works, one can mention, for instance, Laporte (1966), Richardson (1971) and Fry (1988) who studied the potential link between Cubism and the Einsteinian relativity, Fauchereau (1982), Vargish and Mook (1999) or Henderson (2013) who investigated the influence of no-Euclidian geometry on Cubism or Shlain (2007) and Willette (2012) who rather discussed how Cubism, by using neutral tones, dull colors and specific contour drawing would have tampered the expression in painting in accordance with an increasing importance of science in society.

This article aims at contributing to these debates between Cubism and science by focusing on a very specific point: the influence of Cubism on Niels Bohr’s works. So doing, this paper offers a contribution to the history of scientific ideas by proposing an epistemological argument in favour of the assumption made by Miller whereby Niels Bohr has been influenced by a particular form of Cubism (Jean Metzinger) when he developed his non-intuitive complementarity principle.

## Miller’s Assumption on the Visual Dimension of the Principle of Complementarity

In the 1920s, the physicist Niels Bohr understood that the classical visual representation of the atom (a solar metaphor in which the atom’s nucleus acts as the central sun while its electrons move in planetary orbits) was inappropriate. At that time, recent experimental evidence suggested that the atom should simultaneously be seen as a corpuscular and as a wave object. The idea that an electron could be both wave and particle generated much debate and it confused scientists, for whom these two perceptions appear to be ontologically incompatible. Beyond the scientific/ontological debates, this particle-wave duality could also be looked upon as a challenge from a visual (or artistic) point of view. Such a duality was difficult to represent visually because its two components are seemingly in opposition: a particle is often visually represented as a dense body (as a point or a geometric form) while a wave is usually seen as a wavy line or curve.

In 1927, Bohr modified this common way of thinking by proposing his complementarity principle, in which the atomic level is no longer governed by the Newtonian perspective based on interrelated electrons in space and time. This atomic level must instead be analysed using a wave-particle duality framework within which an electron is seen as the sum of its wave and particle properties complementing one another. Depending on the experimental configuration, electrons can exhibit either their wave or particle modes of existence, not both.

How Bohr conceived this complementarity view is sometimes presented as a puzzle in the history of scientific ideas (Miller 2001, p. 395). The complementarity principle can be metaphorically assimilated to psychological studies about the simultaneous existence of reason and emotion and the notion that “we are both onlookers and actors in the great drama of existence” (Favrholdt 1999, p. 40). In this context, several authors have discussed the potential influences of psychology on Niels Bohr’s complementarity principle. For Holton (1970) and Feuer (1974), Kierkegaard, James and Hoffding played an intellectual role in the development of this principle. In the same vein, Pind (2014) explained that Rubin (who was Bohr’s second cousin) also influenced the Danish physicist in his conceptualization of the dual complementarity. It bears mentioning that these influences still generate a lot of debate since Pais (1991), Favrholdt (2009) and Kragh (2012), for example, argued that none of the philosophers previously evoked had any influence on Bohr (see Kragh 2012; or Favrholdt 2009 for further details on this point).

In this article, I will not address these links between simultaneous dualities coming from psychology and the development of the complementarity principle but will rather investigate the visual dimensions which potentially inspired Niels Bohr when he worked on this principle. According to Miller, a part of the solution can be found in the historical influence of cubism on Bohr’s way of thinking. About this influence, Miller (2001) wrote, “The connection with Cubism (Metzinger) is straightforward and not accidental.” However, Miller never explained in details *why* he thought that this influence is straightforward. All contextual information provided by Miller in his remarkable works informed us about *how* a link can be made between Bohr and Cubism but a question is still open: what is the intellectual reason for why one can consider a straightforward link between them? This specific question is at the origin of this article. In different references Miller (1995, 2001) gave historical indications about this relationship between Cubism and Bohr’s works like, for instance, that, in the early 1930s, Bohr moved into a mansion (owned by the Carlsberg Foundation), which he refurbished by buying a Cubist painting (*Woman on a horse*, Metzinger).

**Fig. 1:**
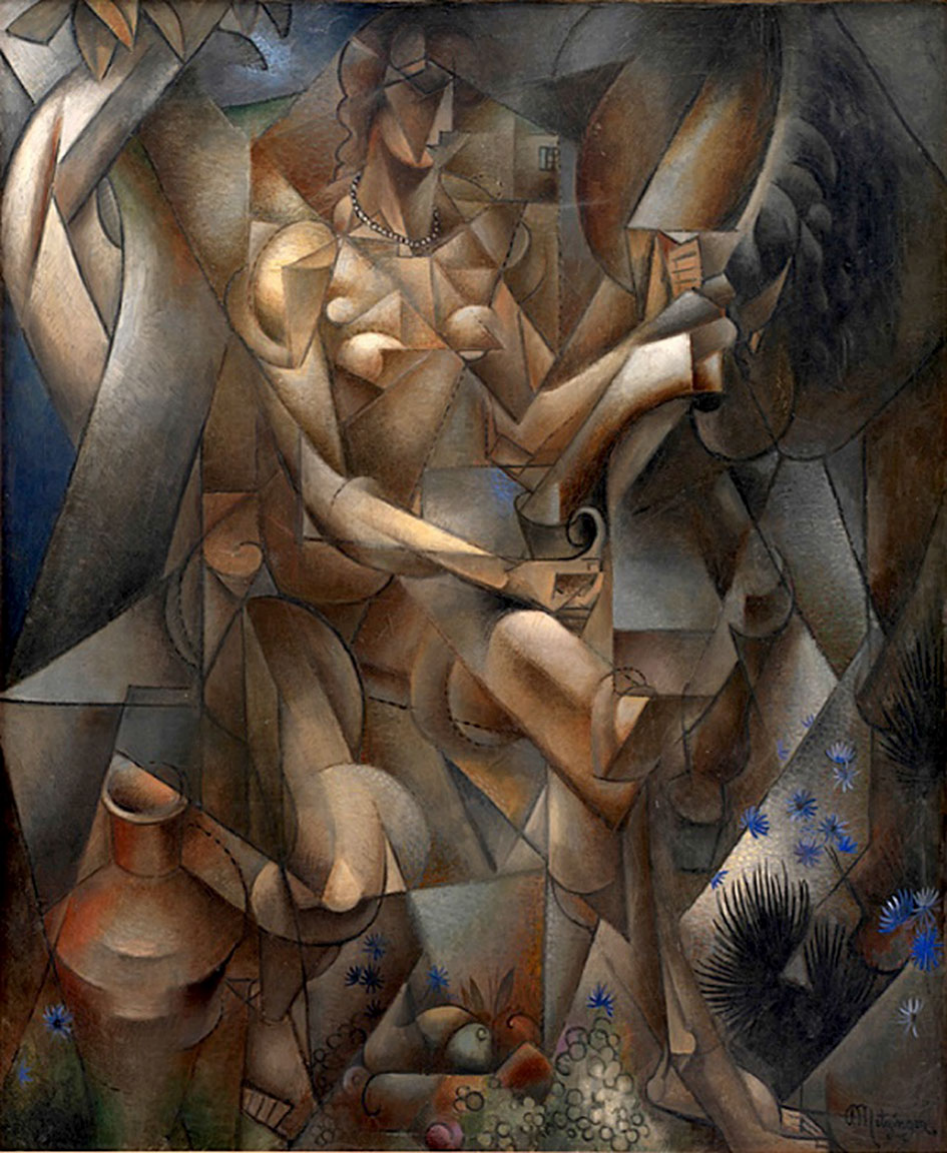
*La Femme au Cheval (Woman with a Horse)*, 1911–1912, Jean Metzinger, oil on canvas, 162 cm × 130.5 cm (63.8 in × 51.2 in), Statens Museum for Kunst

This painting (Fig. 1) represents a woman on a horse in a fuzzy and geometrical juxtaposition of facets breaking the image into a provocative multiplicity of perspectives. Although this painting represents an object that, at first sight, could appeared as fragmented, it rather offers “a representation of visual fragments that are rearranged so that the viewer would not have to move through space in an allotted period of time in order to view them in sequence. Visual segments of the front, back, top, bottom and sides of an object jump out and assault the viewer’s eye simultaneously” (Shlain 2007, p. 189). Combined with the book entitled *Du Cubisme*, this painting is usually presented as a telling example of cubist simultaneity as explained by Fauchereau (1982), Cottington (2004) and Shlain (2007).

The purchase of this painting by Bohr, combined with the testimonies of Mogens Andersen (Bohr’s friend and Danish artist), led Miller to assume that Metzinger had had an influence on Bohr’s way of thinking: “this choice [purchase of the Metzinger’s painting by Bohr] indicates quite an interest in Cubism perhaps [by Bohr], a clue to yet another path to complementarity—that is *assuming* that Bohr knew about Metzinger prior 1927” (Miller 2001, p. 189, my italics). In the following sections, I will investigate this assumption, whose significance goes beyond the mere historical\contextual influence of Metzinger on Bohr. Actually, this plausible influence of Cubism on Quantum Physics goes beyond Miller’s claim since it echoes a vast literature about the epistemic dimension of art and the extent to which art can contribute to an improvement of knowledge (Klinke 2014).

Jean Metzinger (1883–1956) was a French painter, writer and theorist who was initially influenced by neo-impressionism before joining the Cubist movement in 1907. Although Metzinger produced several pieces of art in which he explicitly showed his adhesion to Cubism,^1^ he is nowadays well known for having been at the center of Cubism as one of the first theorist to identify the movement when it first emerged. Metzinger is sometimes presented as a cubist painter “without daring” (Serullaz 1963) who is more famous for his conceptual understanding of the movement that, along with another cubist (A. Gleizes), he summarized in a book entitled *Du Cubisme* (1912). As almost all painters involved in Cubism, Metzinger had several periods in his work: between 1907 and 1913, his works corresponded to what art specialists called “Analytic Cubism” that “describes the early phase of cubism, generally considered to run from 1908 to 1912, characterized by a fragmentary appearance of multiple viewpoints and overlapping plane” (Tate 2016). The major characteristics of this Cubism refers to the rupture with the classical tradition and the importance of the multiple perspectives giving a feeling of simultaneity in the vision (Serullaz 1963). Starting from 1913, Serullaz (1963) explained that Metzinger tried to follow unsuccessfully the Synthetic period of Cubism that mainly corresponded to a systematic used of collages and other materials in works. This evolution showed a more colourful Cubism with simpler lines and shapes introducing some elements of reality in the works. This evolution of Cubism was not really in accordance with what Metzinger was doing and progressively distanced himself from Cubism to focus more on what he called a “realisme constructif” (Metzinger 1972).

In this article will focus on the Metzinger’s Analytic Cubism in order to understand its potential impact on Bohr’s works. There are some works on the Metzinger’s Cubism and its influence on science generated much debates among scholars. Lehrer (2011) wrote, “It is hard to believe that a work of abstract art might have actually affected the history of science. Cubism seems to have nothing in common with modern physics” (this claim could be reinforced by Laporte 1966 or Richardson 1971). These doubts call for an additional argument to support Miller’s perspective. The purpose of this article is to offer an additional argument and to contribute to these debates by providing an epistemological reason for why the association between Metzinger’s Cubism and Bohr’s works is intellectually justified. In the rest of the paper, I will identify what Bohr found in Metzinger’s cubism. That question led me to identify the Bergsonian durée (with its diachronic simultaneity) as the epistemological bridge between Metzinger and Bohr, confirming Miller’s claim according to which Cubism has influenced Niels Bohr in his conception of the complementarity principle.

## From Metzinger’s Simultaneity to Complementarity in Physics

In 1911, room 41 of the Salon d’Automne organized in Paris exhibited a collection of cubist experimentations presented as a rivalry with the futurist paintings (Cottington 2004, p. 74). Although “simultaneity” was a buzzword for both of these artistic movements, they did not use the term to refer to the same concept: futurists mainly painted a visual perception of simultaneity by working on the cinematographic depiction of movement while cubists dealt with a geometric understanding of simultaneity. The futurist simultaneity refers to the simultaneous representation of all subsequent events, but it does not deal with the visual dimensions/perspective of these subsequent events. In contrast, cubists focused on a diachronic simultaneity, in which all visual aspects are holistically captured by juxtaposing all successive aspects of an object in a single pictorial entity. On this point, Vargish and Mook (1999, p. 69) wrote, “When early analysts of Cubism spoke about simultaneity, they meant the representation in cubist art of more than one perspective or of what would represent more than one perspective in the traditional of optical verisimilitude”. This simultaneity is called diachronic simply because this holistic perception of all dimensions is captured as a unique image directly dependent on the observer’s perspective—any physical or temporal changes would therefore generate a new visual representation. According to Cottington (2004), the importance of diachronic simultaneity lies in the visual representation of the Bergsonian durée reasserting temporal continuity/unity. The difference between Cubism and Futurism is clearly explained in the now famous book entitled *Du Cubisme* (1912), written by Metzinger and Gleizes in 1912 who contrasted this sense of simultaneity: “the fact of moving around an object [like Cubists do] to seize it from several successive appearances, which, fused into a single image, reconstitute it in time”—in contrast with—“those [futurists] who mistake the bustle of the street for plastic dynamism will eventually appreciate the differences” (Gleizes and Metzinger 1912, p. 15).

The book *Du Cubisme* played an important role in Miller’s assumption since he assumed that Niels Bohr has read the book before buying Metzinger’s masterpiece: “Since Bohr read widely we can safely assume that he read their [Metzinger and Gleizes] book before 1927.” I claim here that what is important in the potential influence of Metzinger on Bohr’s work is the holistic understanding of the concept of simultaneity promoted by the cubist painter in *Du Cubisme*. By codifying diachronic simultaneity as the “programmatic means [of cubism] to the representation of Bergsonian durée” (Bergson 1911, p. 94), Metzinger opened the door to the idea of complementarity. The Bergsoninan durée refers to a theory of consciousness according to which heterogeneity and continuity of phenomena can be unified in a consistent way. Precisely, the durée can be conceptualized as a unified flow of reality (i.e. unified way of perceiving reality). In this perspective, Bergson considered that space and time are not a void that would be filled by reality. Phenomena are not in space and time but, conversely, the latters are in the former. In other words, time cannot be seen as a multiplicity of moments neither as an abstract eternity—time represents a heterogeneous whole that continually interact with space in a constantly changing flux of renewal. This non-ending and simultaneous repetition of time and space in things can be seen as the Bergsionian durée.

By promoting a holistic perspective where all aspects (even those which are not visible) of an object are depicted in a single image, Metzinger proposed a visual interpretation of Bergsonian simultaneity which refers to the idea that “the real Whole has no parts but is repeated to infinity, each time integrally (though diversely) within itself, and that all these repetitions are complementary to each other” (Bergson 1911, p. 351). The interest of Metzinger in Berson’s simultaneity was evidenced in an article published in 1913 in a daily newspaper (“*Celui qui ignore les* *cubistes*”, G.M, *L’Eclair*, 29th of June, 1913)—this interest has also been evoked by Antliff (1988, p. 341) who wrote “I believe that Du Cubisme was written with Bergson in mind”. The diachronic simultaneity promoted by Metzinger implied a Bergsonian complementarity in which all visual aspects of an object (colours, shadows, perspectives etc.) combine with each other in a complementary but unique cubist image. In this context, cubism can be seen as a visual translation of the unextended durée into an extended and homogeneous multiplicity or juxtaposition of distinct but complementary parts. In line with Bergson’ simultaneity, Metzinger did not promote an absolute simultaneity nor the possibility of having a simultaneity of spatially separated events. Although time, motion and all visual aspects of an object are pictorially fragmented, they are chosen and rearranged by the artist (the observer), permitting the viewer to see them in a single image which is contextually defined: any changes in time or in the observer’s perspective would imply a modification of the image. In accordance with the Bergsonian durée, Metzinger’s simultaneity emerged as a unique event (image) repeated ad infinitum.

According to Miller, the influence of Metzinger on Bohr’s way of thinking has two sources: the purchase of the painter’s masterpiece *Woman with a horse* and a potential reading of *Du Cubisme*. I support this claim by arguing that Bergsonian simultaneity is the epistemological bridge between Metzinger and Bohr. Indeed, when Bohr (1928) defined his principle of complementarity, he implicitly referred to a Bergsonian form of complementarity in which the atomic particle-wave duality must be looked at as a single phenomenon (Lofgren 1992). By considering that an electron can be seen as the sum of its wave and particle properties, which complement one another, the principle of complementarity assumed a diachronic simultaneity (wave and particle at the same time in a single phenomenon). The influence of Bergsonian durée on modern physics is well documented (Guerlac 2006) but what is interesting for this paper is the parallelism between the Bergsonian concept of durée and the principle of complementarity, which, for both Metzinger and Bohr, appears as an epistemic way of understanding a fragmented world. Although all situations are physically composed of a large number of parts, these parts are perceived, at every moment (and ad infinitum), as a complementary unified perception of time (durée). In the same vein, although the universe appears to be fragmented through two incompatible descriptions of matter (particle-wave duality), the complementarity principle proposed by Bohr offers an epistemic way of understanding that complex world as a unified perception at a specific time. This parallelism led Louis de Broglie (1969) to suggest that Bergson anticipated the conceptual framework developed by Bohr. The complementarity principle defined by Bohr implied a Bergsonian durée (with its diachronic simultaneity) that the physicist has possibly found in Metzinger’s works. This influence of the painter on Bohr is enhanced by the idea that, although the perspective (or explanation) given by the observer is presented as a unique image (event), it is directly associated with the angle of observation taken by the observer. As Bohr (Bohr 1928, p. 580) wrote, “in the description of atomic phenomena, the quantum postulate presents us with the task of developing a ‘complementarity’ theory the consistency of which can be judged only by weighing the possibilities of definition and observation”. Regarding the visualization of this theory, the physicist wrote, in accordance with the cubist style, “a more comprehensive generalization of the complementary mode of description will demand a still more radical renunciation of the usual claims of so-called visualization” (Bohr 1937, p. 294). The elements presented here support Miller’s claim that the analytic cubism of Metzinger had a significant influence on the Bohr’s way of thinking.

## Conclusion

In different places, Arthur Miller (1995, 2001) claimed that Metzinger’s Cubism influenced the Bohr’s works. Although Miller gave historical and contextual evidences showing how this influence operated, he did not explain why the association between Cubism and Bohr’s works is intellectually founded. The contribution of this article is to offer an epistemological argument supporting the assumption made by Miller whereby Niels Bohr has been influenced by Cubism (Jean Metzinger) when he developed his non-intuitive complementarity principle. I explained here that the plausible conceptual bridge between Cubism and Quantum Physics is the concept of Bergsonian durée. More precisely, I identified a parallelism between the Bergsonian concept of durée and the principle of complementarity, which appear, for both Metzinger and Bohr, as an epistemic way of understanding a fragmented world: the physicist and the painter considered that, although all situations are physically composed of vast numbers of parts, these parts are perceived, at every moment (and ad infinitum), as a complementary unified perception of time (durée).

This article is a contribution to the field of history of scientific ideas in which there is, unfortunately, very few historical (physical) evidences. By definition, the history of ideas implies another “proof regime” which is rather based on what philosophers of science call a “rational reconstruction a posteriori” (Lakatos 1978). This paper is a telling example of such as rational reconstruction that refers to a combination of plausible historical facts (enhanced by Arthur Miller) with a rational\logical reasoning (proposed in this paper). Beyond the contribution to the history of scientific ideas evoked above, this short paper also emphasizes that art can also play a key role in the development of human knowledge. Indeed, while one can find a lot of articles on how art is influenced by science, this paper suggests the reciprocal link since science can also be influenced by art.

## Footnotes

1 See Serullaz (1963) or Robbins (1985) for an exhaustive list of Metzinger’s paintings.
